# From opt-out to opt-in consent for secondary use of medical data and residual biomaterial: An evaluation using the RE-AIM framework

**DOI:** 10.1371/journal.pone.0299430

**Published:** 2024-03-28

**Authors:** Jennifer E. Lutomski, Peggy Manders

**Affiliations:** 1 Radboud Biobank, Radboud University Medical Center, Nijmegen, The Netherlands; 2 School of Allied Health Professionals, Fontys University of Applied Sciences, Eindhoven, The Netherlands; University of Oxford, UNITED KINGDOM

## Abstract

**Background:**

Patient records, imaging, and residual biomaterial from clinical procedures are crucial resources for medical research. In the Netherlands, consent for secondary research has historically relied on opt-out consent. For ethical-legal experts who purport passive consent undermines patient autonomy, opt-in consent (wherein affirmative action is required) is seen as the preferred standard. To date, there is little empirical research exploring patient feasibility, organizational consequences, and the potential risks for research based on secondary data. Thus, we applied the RE-AIM framework to evaluate the impact of migrating from an opt-out to an opt-in consent process.

**Methods:**

This evaluation was carried out in Radboud University Medical Center, a large tertiary hospital located in the southeast of the Netherlands. All non-acute, mentally competent patients ≥16 years of age registered between January 13, 2020 and June 30, 2023 were targeted (N = 101,437). In line with the RE-AIM framework, individual and organizational consequences were evaluated across five domains: reach, efficacy, adoption, implementation, and maintenance.

**Results:**

101,437 eligible patients were approached of whom 66,214 (65.3%) consented, 8,059 (7.9%) refused consent and 27,164 (26.8%) had no response. Of the 74,273 patients with a response, 89.1% consented to secondary use. The migration to an opt-in consent system was modestly successful; yet notably, differential response patterns by key sociodemographic characteristics were observed. Adaptions to the process flow improved its effectiveness and resulted in a reasonable response over time. Implementation was most affected by budgetary restraints, thus impeding the iterative approach which could have further improved domain outcomes.

**Conclusion:**

This evaluation provides an overview of logistical and pragmatic issues encountered when migrating from opt-out to opt-in consent. Response bias remains a major concern. Though not always directly transferable, these lessons can be broadly used to inform other health care organizations of the potential advantages and pitfalls of an opt-in consent system.

## Background

Patient records, imaging, and residual biomaterial from clinical procedures are crucial resources for medical research. Yet, consent processes for accessing these data and biomaterial vary across the European Union, with some countries practicing opt-in consent and others opt-out [1–3]. This poses an ethical incongruity since opt-in consent requires affirmative action from a patient whereas opt-out consent assumes consent unless otherwise stated. In the Netherlands, consent for secondary research has historically relied on the opt-out method. This passive consent process was deemed ethically acceptable provided that patients are adequately informed about potential secondary use. Moreover, a clear and simple withdrawal procedure is required.

Disconcertingly, however, these two conditions have not always been achieved in practice. An unpublished quality control assessment carried out in the Radboud University Medical Center (Radboudumc, Nijmegen, the Netherlands) found that approximately nine in ten patients were unaware of secondary use and consequently the option to withdraw consent. This sub-optimal outcome was likely due to organizational structures leading to limited visibility of relevant information. Dutch professional working groups, comprised of representatives from university medical centers as well as smaller local hospitals, have acknowledged that this observation is not an isolated finding.

Lack of awareness surrounding secondary use is further compounded by the current overarching ethical and legal climate, which harbors concerns that opt-out consent undermines patient autonomy [4–6]. Still, it is important to emphasize that most patients are generally receptive to secondary use of their data and residual biomaterial as long as they are informed [7–9]. Dutch patients are no exception [10, 11].

Given this backdrop, there is a clear and inherent need to better inform patients about secondary use for medical research, either via informed opt-out or opt-in consent. However, to date, there is little empirical research exploring the feasibility for patients, organizational consequences, and the potential risks for researchers who rely on secondary use. To address this confluence of factors, we applied the RE-AIM framework to evaluate the feasibility of migrating from an opt-out to an opt-in consent process.

Briefly, whereas many standard methods exist for evaluating the effectiveness of an intervention or policy change, often these measures do not address robustness, translatability and/or public health impact [12]. The RE-AIM framework was developed to bridge this gap and serve as a broadly applicable tool to evaluate the individual and institutional impact of an intervention or policy across five domains: Reach, Efficacy, Adoption, Implementation, and Maintenance [13, 14]. By drawing stakeholders attention toward these five essential elements, new initiatives can be systematically assessed [12]. This not only brings rigor to the evaluation process but also allows for comparability [14]. Since its inception in 1999 [14], the RE-AIM framework has been widely used in a variety of health care settings [12]. It has subsequently been deemed a robust tool to evaluate the sustainability of new initiatives in practice. This framework is particularly useful in the context of this study since it includes both individual- and institutional-level impact, thus encompassing the interwoven complexities which would likely be obscured if only one perspective were applied [12].

Thus, the primary goal of this study was to implement and evaluate a hospital-wide opt-in consent process for the secondary use of patient records, imaging, and residual biomaterial according to the RE-AIM framework.

## Methods

### Setting and population

This study was carried out in the Radboudumc, a large (~1,000 beds) tertiary hospital located in the southeast of the Netherlands. Notably, previous research into the use of opt-in consent in the Netherlands was conducted in a cancer treatment and research hospital (Antoni van Leeuwenhoek Hospital, Amsterdam) [15]. When opt-in consent became the standard policy in this hospital, response was very high (>90%), with minimal refusals. Yet, cancer patients have been historically more receptive to secondary use of data [7, 11]. To determine if such findings would translate to a general patient population, Radboudumc was chosen as one of the first feasibility test sites given its status as a university medical center serving a diverse patient case-mix.

Radboudumc currently maintains over 2.3 million patient files in Epic (Verona, Wisconsin, USA), which is an electronic medical records systems. Approximately 500 new patients are registered every week. Given the enormity of the existing patient population, retrospective opt-in consent was not performed. Therefore, this study targeted all new patients as of January 13, 2020. A new patient was defined as an individual who was not previously registered in the hospital. A final evaluation was undertaken on June 30, 2023. This timeframe was chosen as it would allow the assessment of approximately 100,000 patients and ample time to monitor trends.

Vulnerable subpopulations, specifically acute care, mentally incompetent, comatose, and minor (<16 years old) patients, were excluded from this evaluation. Consent protocols for these subgroups would require special adaptations to adhere to legal and ethical requirements. Notably, Dutch statute law recognizes legal adulthood for medical decision-making from 16 years of age and older.

Given that anonymized, aggregated sociodemographic patient data were extracted, this evaluation falls outside the remit of the Medical Research Involving Human Subjects Act (in Dutch: *Wet Medisch-wetenschappelijk Onderzoek met mensen*, WMO). Therefore, this research was permitted to be conducted in the Netherlands without additional approval by an accredited research ethics committee [CMO Radboudumc; Project Title: Consent at the Gate (in Dutch: *Toestemming aan de Poort*); File number: 2019–5396]. It is important to clarify that this is the verbatim ethics statement recommended for publication by the local Committee on Research Involving Human Subjects (in Dutch: CMO, *Commissie Mensgebonden Onderzoek*). In the Netherlands, research is only reviewed by an accredited medical research ethics committee if it falls under the scope of the Medical Research Involving Human Subjects Act [16]. This type of research, often referred to ‘WMO’ research, has two criteria: “(a) concerns medical scientific research and (b) participants are subject to procedures or are required to follow rules of behavior” [16]. In Radboudumc, the local Committee on Research Involving Human Subjects conducts an expedited review to determine whether or not these criteria are met.

### General protocols: Opt-out versus opt-in consent

An opt-out consent protocol was in effect in Radboudumc until the start of the feasibility study. Prior to 2015, patient information on secondary use, including the procedure to withdraw consent, was available on-site as a hard copy pamphlet. To withdraw consent, patients would have to inform their health care practitioner. In 2015, a patient portal account, henceforth referred to as *mijnRadboud*, was introduced. Hard copy pamphlets were discontinued; they were replaced with a webpage. Abridged patient information was available in *mijnRadboud* along with an opt-out button to simplify the consent withdrawal process. A link to the webpage hosting the unabridged patient information was also available. In addition to *mijnRadboud*, patients could still withdraw consent via their health care practitioner.

As of January 13, 2020, all new patients were prospectively approached to consent to secondary use of their patient records and residual biomaterial for medical research. Prior to visiting the hospital, patients received a referral letter which stated their appointment details as well as instructions on how to activate their personal *mijnRadboud* account. Identity verification, which is conducted during patient registration, is required for full access to *mijnRadboud*. However, after activating their account, patients had access to a restricted environment in which they could read patient information on secondary use as well as review the consent question. The consent question was stated as follows:

*In order to properly examine and treat you*, *we record your medical information*, *and sometimes we collect biomaterial such as blood*, *urine or tissue*. *In some cases*, *Radboudumc would like to use your medical information and leftover biomaterial for scientific research*. *This would support research into improved treatments and health care outcomes*. *Do you consent to the use of your medical data and residual biomaterial for research*?

During registration on the day of their scheduled appointment, an administrative assistant would confirm whether the consent question was completed in *mijnRadboud*. If not, the administrative assistant would give a brief background and pose the aforementioned consent question. A plain language infographic was available as a visual aid to assist in the explanation. All administrative assistants were trained to inform patients in plain language that secondary use only pertains to research with a low chance of incidental findings and could not be used for research involving embryos, a fetus, and/or with gametes used to create embryos. They were also trained to inform patients that researchers would only be allowed access to pseudo-anonymized data that are not directly traceable back to the individual patient. Patients would orally consent or refuse, and this was immediately recorded in their patient file in EPIC. If further deliberation time was required, the patients would receive a card with the link to the webpage. Via the webpage, patients could read more information about secondary use written in plain language Dutch or English. The webpage describes common patient concerns and questions regarding secondary use. They could also submit questions and/or request to be contacted via telephone.

Over the study period, patients with no response for the consent question were sent two reminders. This reminder was sent to their registered email address via *mijnRadboud*. Email reminders were chosen since approximately 85% of patients use either the restricted or full-access *mijnRadboud* environment.

### Applying the RE-AIM framework

#### Reach

Reach is defined as “the absolute number, proportion, and representativeness of individuals who are willing to participate in a given initiative, intervention, or program” [13]. To assess this dimension, we analyzed the proportion of response (either consent or refusal) versus non-response. In line with generally accepted thresholds, our response target was ≥75%. To examine representativeness, we extracted key demographic characteristics, namely age, gender, clinical department visited, and country of origin for both responders and non-responders. These characteristics were chosen since there were the most likely to be completed in the electronic medical record with reliable results. Notably, country of origin does not distinguish the number of years in the Netherlands, and thus presumably this variable includes a heterogenous group of patients with differing proficiencies in Dutch language and knowledge of the local medical system. Due to large numbers, statistical testing across demographic groups was not carried out.

#### Effectiveness

Effectiveness is defined as “the impact of an intervention on important outcomes, including potential negative effects, quality of life, and economic outcome, [including] heterogeneity of effects and reasons for success or lack of such” [13]. Since the change in the consent procedure was expected to bring minimal risk to patients who responded to the consent questions, our examination of effectiveness largely focused on non-responders. During the first phase of the evaluation (January 20, 2020 to March 31, 2020), telephone contact was made with non-responders to administer consent and gain information regarding underlying reasons for non-response, be it intentional (i.e., unclear information) or unintentional (i.e., missed in the process flow). To reduce patient burden, this was not intended to be an in-depth qualitative undertaking but rather a broad oversight into underlying reasons for non-response. Feedback was collected and completely anonymized and therefore cannot be linked to individual patients. This telephone contact was conducted as part of quality assurance within the hospital.

#### Adoption

Adoption is defined as “the absolute number, proportion, and representativeness of a) settings; and b) intervention agents (people who deliver the program) who are willing to initiate a program” [13]. In Radboudumc, there are approximately 35 rotating medical administrative assistants responsible for administering the consent question and updating the patient’s consent status in EPIC. Thus, to evaluate this domain, we administered a short on-line questionnaire to all administrative assistants using Castor EDC [17] between June 1, 2022 and June 30, 2022. An informational email preceded the survey to encourage response; one reminder was also sent one week after the initial survey invitation. In brief, the de-identified survey covered the general awareness of the new consent procedure; frequency of administering the consent question; underlying reasons (if applicable) for not administering the consent question; suggestions for improved support; general barriers in implementation; perceived importance of consent question; and number of years employed by Radboudumc. This survey was conducted as part of quality assurance within the hospital.

#### Implementation

At the setting level, implementation is defined as “the intervention agents’ fidelity to the various elements of an intervention’s protocol, including consistency of delivery as intended and the time required…[it] also includes adaptations made and the costs of implementation”. On the individual level, it “refers to clients’ use of the intervention and implementation strategies” [13]. For the setting-level evaluation, we provided a narrative description of organizational and logistical obstacles encountered during the implementation process and implemented solutions.

#### Maintenance

Maintenance is defined as “the extent to which: a) behavior is sustained six months or more after treatment or intervention; and b) a program or policy becomes institutionalized or part of the routine organizational practices and policies” [13]. Due to the large organizational changes that were required, a longer evaluation period was adopted. A narrative description on the current practice and policy is provided. We further examined response trends in patients between January 13, 2020 and June 30, 2023.

## Results

### Reach

Over the evaluation period, there were 101,437 eligible patients of whom 66,214 (65.3%) consented, 8,059 (7.9%) refused consent and 27,164 (26.8%) had no response. Thus, a 73.2% total response (consent/refuse consent) was achieved. Of the 74,273 patients with a response, 89.1% consented to secondary use of their medical data and residual biomaterial for research purposes.

No notable differences in consent status were observed across gender, and this observation was stable over time (Table 1). However, differences were observed across age groups. Consent was highest between ages 40 and 69. Response was markedly lower in the oldest age group (≥80 years) and modestly lower for patients aged 16 to 39 years. Expectedly, the vast majority of patients were born in the Netherlands. Relative to patients born outside the Netherlands, Dutch patients were not only more likely to respond but also to consent to secondary use. Variation in response patterns were observed between common migrant population in the Netherlands. For example, whereas patients born in Indonesia or the United Kingdom were more likely to consent, patients from Morocco and Turkey were more likely to refuse consent. Non-response was disproportionately higher for patients with an unknown country of birth.

**Table 1:** Sociodemographic distribution of consent status for secondary use of data and residual biomaterial for research (N = 101,437).

		Consent	Refused consent	No response
	Total (N)	n (%)	n (%)	n (%)
**Gender**				
Man	45,403	29,888 (66)	3,117 (7)	12.398 (27)
Woman	56,008	36,308 (65)	4,938 (9)	14,762 (26)
Unspecified/other	26	-^1^	-^1^	-^1^
**Age in years**				
16–19	4,330	2,600 (60)	403 (9)	1,327 (31)
20–29	16,023	9,604 (60)	1,545 (10)	4,874 (30)
30–39	17,043	10,290 (60)	1,859 (11)	4,894 (29)
40–49	11,961	7,889 (66)	1,128 (9)	2,944 (25)
50–59	17,353	12,204 (70)	1,226 (7)	3,923 (23)
60–69	17,851	12,814 (72)	1,039 (6)	3,998 (22)
70–79	12,890	8,652 (67)	629 (5)	3,609 (28)
≥80	3,986	2,161 (54)	230 (6)	1,595 (40)
**Country of origin**				
Netherlands	75,219	55,318 (74)	6,130 (8)	13,771 (18)
Dutch Caribbean^2^	304	182 (60)	40 (13)	82 (27)
EU member states^3^	2,532	1,604 (63)	208 (8)	720 (28)
Select other countries^4^				
China	167	103 (62)	31 (19)	33 (20)
Indonesia	405	288 (71)	39 (10)	78 (19)
Morocco	376	169 (45)	94 (25)	113 (30)
Suriname	282	171 (61)	46 (16)	65 (23)
Syria	506	223 (44)	66 (13)	217 (43)
Turkey	768	367 (48)	153 (20)	248 (32)
United Kingdom	180	134 (74)	12 (7)	34 (19)
Unknown	17,915	6,125 (34)	881 (5)	10,909 (61)

^1^The consent distribution for unspecified/other gender is not shown due to small numbers.

^2^The Dutch Caribbean, which falls under the Kingdom of the Netherlands, refers to the countries Aruba, Curaçao, and Saint Maarten as well as the municipalities Bonaire, Saint Eustatius, and Saba.

^3^EU member states include Austria, Belgium, Bulgaria, Croatia, Cyprus, Czechia, Denmark, Estonia, Finland, France, Germany, Greece, Hungary, Ireland, Italy, Latvia, Lithuania, Luxembourg, Malta, Netherlands, Poland, Portugal, Romania, Slovakia, Slovenia, Spain and Sweden.

^4^Selected countries represent several of the commonest immigrant populations in the Netherlands.

Differences were observed across medical departments. When reviewing the ten most frequently visited departments (Table 2), non-response was highest for the Department of Genetics and the Department of Obstetrics and Gynecology. Consent refusal was notably higher in the Department of Fertility and Reproductive Health.

**Table 2:** Distribution of consent status for secondary use of data and residual biomaterial for research by most frequently visited medical department.

	Number of patients	Consent	Refused consent	No response
Department	N	n (%)	n (%)	n (%)
Neurology	5,769	4,386 (76)	411 (7)	972 (17)
Obstetrics and Gynecology	5,401	2,658 (49)	473 (9)	2,270 (42)
Genetics	5,153	2,605 (51)	289 (6)	2,259 (44)
Ophthalmology	4,075	2,539 (62)	335 (8)	1,202 (29)
Dermatology	4,036	2,802 (69)	318 (8)	916 (23)
Oral and Maxillofacial Surgery	3,975	2,415 (61)	384 (10)	1,176 (30)
Fertility and Reproductive Health	3,764	2,324 (62)	634 (17)	806 (21)
Internal Medicine	3,321	2,367 (71)	294 (9)	660 (20)
Otorhinolaryngology (ENT)	1,709	1,156 (68)	138 (8)	415 (24)
Urology	1,648	1,159 (70)	163 (10)	326 (20)

### Effectiveness

Between January 20, 2020 and March 31, 2020, records were extracted from 700 patients with no response for the consent question. Of these, 316 patients consented and 47 refused consent. There were two main themes that arose from telephone contact: a) patients were either missed/unaware of the consent question or b) patients believed they had already consented to secondary use.

Several administrative hurdles were observed. After three contact attempts, 72 patients were unable to be reached. Furthermore, 201 patients did not have a contact telephone/mobile number recorded in EPIC, 43 had an inactive number, and four had an incorrect number. There were several other minor reasons why consent could not be administered. These were grouped together due to small numbers (n = 17) and were as follows: a) did not want to complete the consent question over the telephone; b) mentally incompetent and erroneously selected in the data extraction due to a coding error; c) language barrier; d) death over evaluation period. This feedback was used to improve the process flow of routine administrative operations in the hospital.

#### Adoption

Fifteen of the 35 medical administrative assistants replied to the on-line questionnaire (response: 42.8%). Most (n = 12) reported always or almost always administering consent. The most commonly cited reason for not administering consent was failing to remember. Medical administrative assistants who were employed less than one year were more likely not to administer the consent question. An additional prompt in Epic and enhanced visual aids were suggestions to improve the consent procedure. The most commonly reported obstacles were patients with specific/difficult questions and limited examples of secondary use in current research. Nearly all strongly agreed or agreed (n = 12) that the consent question was important for research and improving care in the hospital.

#### Implementation

Restricted funding remained an overarching implementation issue during the evaluation period. One 0.2 FTE (full-time equivalent) employee was responsible for the coordination of the initiative, training, interim evaluations, and communication. In the ideal situation, feedback from patients and administrative assistants would be re-administered at multiple timepoints to permit an iterative approach to adaptions. Moreover, mixed method research into reasons for non-response or refusing consent could have guided improved patient communication. However, these additional measures were unachievable within the allotted budget. Funding directly impacted the administration of patient reminders. Whereas most reminders could be sent via email for completion via *mijnRadboud*, a subset of patients with no response (approximately 15,000) did not use either the restricted or full-access *mijnRadboud* environment. Budget and time constraints related to materials/postage, data entry, and storage inhibited mailing hard copy consent forms.

Several adaptations were implemented based on findings from the telephone contact (see Effectiveness section) and the feedback from the administrative assistants (see Adoption). Greater visibility was given to the consent question in *mijnRadboud* to encourage patients to complete the question prior to their visit. This was done by linking the consent question with other preparatory questions, such as verifying their address, phone number, and primary care physician. The website was adapted to give extra information, and additional training support was provided to the administrative assistants.

Given the level of non-response from the initial start of opt-in consent procedure, two additional reminders were mailed. In absolute terms, the overall response increased by at least 3% per reminder. It is important to emphasize, however, that there was an substantial increase in patient inquiries following each reminder. Whereas the Radboudumc general help desk generally receives 50 phone inquiries per day, this increased to 300 the day following the reminder. This was a short-term though marked burden for the help desk.

#### Maintenance

Due to the adaptions undertaken to the process flow as outlined in the section ‘Implementation’ in the first nine months of the evaluation, response increased from 47% (8,860 responders / 18,747patients approached) in September 2020 to 73% (74,267 responders / 101,430 patients approached) in June 2023. This response pattern was fairly stable over the last six months of the evaluation, suggesting that the initiative has become well integrated into the routine process flow of the hospital.

**Fig 1:**
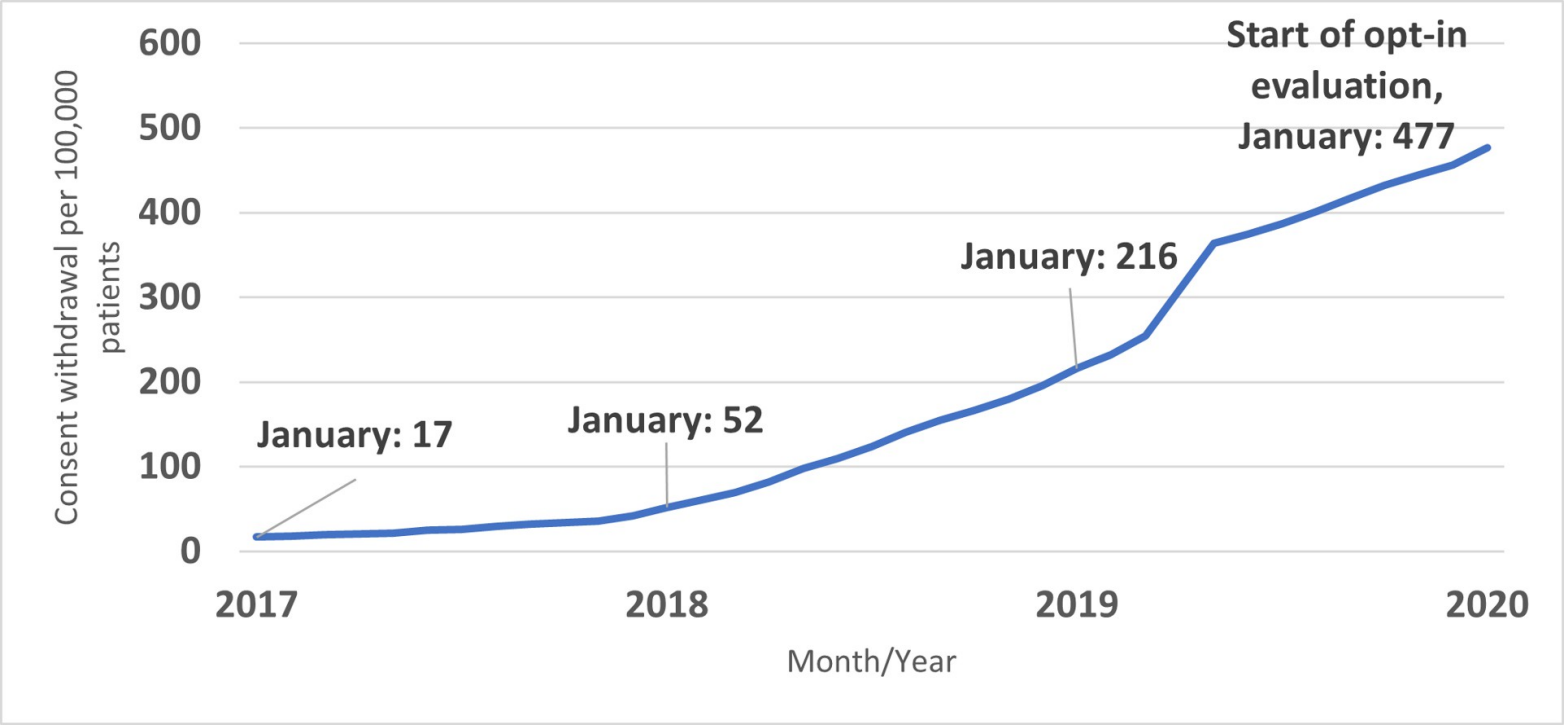
Rate of consent withdrawal under opt-out consent per 100,000 patients, Radboud University Medical Center, January 2017-January 2020.

Trends in consent refusals were consistent over the evaluation period and remained at 11% even when the overall response improved. Thus, it appears that there is a specific proportion of the patient population who will steadily refuse consent for secondary use. However, the marked disparity in the proportion of consent refusals by consent procedure should be noted. In contrast to opt-in consent, <1% of patients withdrew consent under the opt-out regime. Still, even before the transition to opt-in consent, an increasing trend in consent refusal was observed. This occurred after the introduction of *mijnRadboud* (Fig 1). Thus, *mijnRadboud* likely serves as a critical patient information point which supports informed decision-making under both opt-out and opt-in consent protocols. It further played an integral role in the maintenance of the opt-in initiative.

## Discussion

Based on the RE-AIM framework, the migration from an opt-out to an opt-in consent system in a large tertiary hospital was moderately successful. Adaptions to the process flow improved its effectiveness and resulted in a reasonable response in the context of typical research recruitment goals. In general, administrative assistants found the consent question meaningful and important for patient care. Their willingness to adopt the new initiative has supported its long-term maintenance. Most critical, however, we did not meet our 75% response threshold differential and response patterns differed by key sociodemographic characteristics. Speculatively, response could have been improved; yet, implementation was hindered by budgetary restraints, impeding an iterative approach which could have further improved domain outcomes.

To the knowledge of the authors, this is the largest study examining opt-in response for secondary research. A recent systematic review pooled 13 studies to achieve a similar sample size [18]. Reassuringly, consent among patients who responded in our evaluation was in line with these pooled results, which reported an average weighted consent of 84% [18]. Still, whereas this review observed that men were more likely to consent, no marked gender differences were observed in our evaluation.

There were notable demographic patterns in non-response, with older and younger patients less likely to respond. Barriers in recruiting older persons are widely recognized in the literature [19]. Increased visual/auditory impairments and/or mild cognitive decline may have not only deterred use of *mijnRadboud* but also hindered oral administration of the consent question. Targeted training and visual aids could improve response outcomes in older subgroups.

However, we were unable to discern why non-response was modestly higher in patients aged between 16 and 39 years. This may be an artefact of generational differences in the patient population. To date, there has been robust research on minors (<18 years), which has shown that younger age [20, 21] and having less disease experience [20] are associated with decreased interest in research participation. Speculatively, younger patients may then feel less compelled to complete the consent question. Moreover, the inherent nature of secondary research may have served as a response deterrent since younger persons are less likely to participate in low-risk, less-complex research [21]. Parents/guardians, whose perspectives may be influenced by the potential for enhanced care [22–24], likely played an instrumental role in response patterns as well. Although parental consent is not required from the age of 16 years and older, it is not unreasonable to envision that these younger patients were typically accompanied by their parents/guardians. Still, given that lower response was not restricted to minors, further research into factors influencing response patterns in patients aged between 18 and 39 years may be warranted. This would likely require a qualitative or mixed-method approach.

The number of patients with an unknown country of birth was relatively high since it is a non-mandatory field in the electronic patient record. Length of residence in the Netherlands could not be determined, limiting the ability to use country of birth as a proxy for language level or knowledge of the Dutch healthcare sector. Using available data, our evaluation did suggest that cultural differences in consent patterns are present. We were, however, unable to discern if these differences arose due to languages barriers, varying cultural receptiveness toward secondary research, or a combination of both. Upon the initiation of this study, information was only available in Dutch and English. This may have been an influencing factor for higher refusals and non-response in residents who were not fluent in these languages. The findings from this study did prompt Radboudumc to ultimately translate the secondary use infographic into plain language German, Polish, Ukrainian, Turkish, and Arabic to aid administrative assistants in communicating with common immigrant groups. Ideally, cultural differences in response would continue to be monitored to evaluate what effect, if any, these translations had. Additional culturally competent patient information to improve response outcomes may be warranted.

An important theme that arose to evaluate effectiveness is the marked number of non-responders who believed they had already consented to secondary use. This observation was unsurprising given that new patients are often confronted with numerous consent requests in a research hospital. Patients are asked to consent to electronic sharing of medical data between their care providers, auditing for quality control measures, and individual research studies. This can elicit consent confusion for patients. Although efforts have been undertaken with the Patient Communication Department, this remains a pervasive issue. It is unclear if this misconception decreased the effectiveness of the email reminders. Potentially patients could be less likely to react to the email reminder if they deemed it redundant.

The evaluation period coincided with the COVID-19 pandemic, which largely impacted the implementation of the initiative. For instance, internal and external communications were shifted to high-priority COVID-related updates. This likely contributed to the lower response observed in the early phase of the evaluation. Moreover, if possible, patients were re-routed to video consultations during the pandemic. Response disparities on the departmental level may have been due to this reconfiguration of the administrative process flow. The Department of Genetics, which conducted the largest proportion of video consultations, also witnessed a higher proportion of non-response.

Still, not all departmental response patterns can be attributed to the pandemic. The Department of Reproductive Medicine reported the highest proportion of patients who refused consent. Patients in this care pathway were likely concerned about the use of their gametes and/or embryos for scientific research. Yet, this type of research falls under the Dutch Embryo Act (in Dutch: *Embryowet*) [25] and therefore would be excluded from consent for secondary use. In light of this observation, the training manual script was adapted in an attempt to better inform patients that certain types of research do not fall under this broad opt-in consent. Yet, given that the high proportion of refusal persisted over the evaluation period, it appears this information is difficult to convey.

Opt-in versus opt-out consent for secondary use remains a controversial topic. To date, there is no national consensus in the Netherlands. Proponents for opt-in consent typically voice ethical concerns about patient autonomy and transparency. These concerns may be valid given the disparity between consent refusal under an opt-out versus opt-in consent protocol. Prior to the introduction of opt-in consent in Radboudumc, less than 1% of patients withdrew their (passive) consent. This is in stark contrast to the nearly 11% of patients who refused consent when actively approached. We were unable to determine why this disparity occurred. It may be due to patients being better informed, but alternatively, it may also be due to patients misunderstanding the consent question or time constraints. This is a key distinction because if a large proportion of refusals stems from implementation issues rather than the patient’s own conscious volition, this would bolster the argument to revert to an informed opt-out consent system. Further research investigating underlying reasons for refusing consent would help direct future initiatives. Specifically, mixed method research would greatly enhance our current understanding of the underlying dynamics which result in refusal for secondary use. This type of research would be useful in cases of non-response as well.

Patient concerns over data security and privacy may have potentially impacted the frequency of refusals or non-response [11]. Patients generally understand that secondary use promotes scientific research and innovation, but pervasive fears of misuse can hinder their receptiveness to data reuse [11]. In this context, it may be useful to inform patients how cloud computing and virtualization have allowed for the development of digital research environments. These settings create secure and auditable virtual workspaces and do not require data transfers between local hard drives [11, 26, 27]. Better understanding of digital security may have further improved response. Given that the Dutch population (along with the Finnish) has reported the highest level of overall digital skills in the European Union, this would likely facilitate conversations regarding digital research environments [28].

Medical administrative data are a critical source for research. Arguably, some may view a 75% response too low of an acceptable threshold for secondary use. Depending on the research question and the variation of response in select patient subgroups, even minor refusal and/or non-response could result in major bias issues or inhibit certain research studies altogether. Identifying a universal solution to bypass such issues is challenging since the direction and magnitude of bias is intrinsically linked with the research question. Whereas some research questions may be heavily biased as a result of opt-in consent, this is not necessarily the case for all research questions. Still, as evidenced in our evaluation, opt-in consent would likely heighten the risk of response bias for certain research questions due to non-response (e.g. older populations) and demands substantial organizational requirements to ensure these populations are adequately sampled. If opt-in is selected as the preferred method of consent, a thorough evaluation of mechanisms to mitigate potential bias would be necessary.

Notably, research is currently underway in Radboudumc to evaluate opt-in consent in acute, mentally incompetent, comatose, and minor (<16 years old) patients. These vulnerable subgroups may be even more likely to refuse or not respond to the consent question. Yet, for these groups in particular, the ethical dynamics must balance both patient autonomy and societal benefits. For instance, for acute and comatose patients, a consent waiver or emergency exception likely undermines autonomy and is not suitable in many cases [29]. Yet, patient proxies may be influenced by the urgent nature of the clinical setting and/or therapeutic misconception (i.e. the notion that secondary research would have a direct benefit for the individual patient) [29]. This may, in ethical terms, undermine the validity of the consent. For mentally incompetent patients, ethical and legal spheres concur that incapacity to give informed consent for one medical intervention does not automatically preclude the ability to consent for another [30]. Thus, consent for secondary use may also need to be viewed through this lens. Lastly, minor patients may be encouraged or discouraged from consenting to secondary use [31], thus it is critical that information adequately addresses the needs and concerns of both parties.

Although our findings suggest that opt-in consent is achievable in practice, not all health care institutions have the same available resources and infrastructure. Compared to the patient populations in other Dutch hospitals, a markedly larger proportion of Radboudumc patients (approximately 85%) use an electronic patient portal (results not published). This was likely linked to the success of our evaluation. If other Dutch hospitals are not able to achieve the successes observed in the Radboudumc, this could reasonably be an argument against opt-in consent since it would create disjointed response patterns across the country.

It is also important to underscore that the proposed Dutch Control over Human Biomaterials Act (in Dutch: *Wet Zeggenschap Lichaamsmateriaal*) and the General Data Protection Regulation (GDPR, Regulation 2016/679) [32] do not preclude the use of opt-out consent for secondary research. Moreover, as the European Parliament strives to create a European Health Data Space to facilitate international data sharing, opt-out consent for the secondary use has been deemed legal and ethical [33]. Developments in this area remain on-going in the European Union, and consequently local Dutch procedures may be driven by European policies.

In conclusion, this evaluation provides an overview of logistical and pragmatic issues encountered by a large tertiary hospital migrating from opt-out to opt-in consent. Response bias remains a concern. Though not always directly transferable, these lessons can be broadly used to inform other health care organizations of the potential advantages and pitfalls of an opt-in consent system.
